# Impact Strength and Water Uptake Behavior of Bleached Kraft Softwood-Reinforced PLA Composites as Alternative to PP-Based Materials

**DOI:** 10.3390/polym12092144

**Published:** 2020-09-20

**Authors:** Helena Oliver-Ortega, Quim Tarrés, Pere Mutjé, Marc Delgado-Aguilar, José Alberto Méndez, Francesc Xavier Espinach

**Affiliations:** 1 Laboratori d’Enginyeria Paperera i Materials Polímers (LEPAMAP Research Group), Universitat de Girona, Campus Montilivi, C.P. 17003 Girona, Spain; helena.oliver@udg.edu (H.O.-O.); pere.mutje@udg.edu (P.M.); m.delgado@udg.edu (M.D.-A.); jalberto.mendez@udg.edu (J.A.M.); 2 Càtedra de Processos Industrials Sostenibles, Universitat de Girona, Campus Montilivi, C.P. 17003 Girona, Spain; 3 Design, Development and Product Innovation, Dept. of Organization, Business, University of Girona, 17003 Girona, Spain; francisco.espinach@udg.edu

**Keywords:** natural fibers, impact behavior, mechanical testing, injection molding, water uptake

## Abstract

The research toward environmentally friendly materials has devoted a great effort on composites based on natural fiber-reinforced biopolymers. These materials have shown noticeable mechanical properties, mainly tensile and flexural strengths, as a consequence of increasingly strong interfaces. Previous studies have shown a good interface between natural fibers and poly (lactic acid) (PLA) when these fibers present a low lignin content in their surface chemical composition (bleached fibers). Nonetheless, one of the main drawbacks of these materials is the hydrophilicity of the reinforcements in front of the mineral ones like glass fiber. Meanwhile, the behavior of such materials under impact is also of importance to evaluate its usefulness. This research evaluates the water uptake behavior and the impact strength of bleached Kraft softwood-reinforced PLA composites that have been reported to show noticeable tensile and flexural properties. The paper explores the differences between these bio-based materials and commodity composites like glass fiber-reinforced polypropylene.

## 1. Introduction

The research in bioplastics has increased during the past years because of environmental awareness. Biopolymers are obtained from renewable resources such as plants or bacteria and represent a sustainable alternative to oil-based polymers. The scarce of oil and the high dependency of the worldwide population has increased the interest of researchers and industry in biopolymers [1,2,3]. Although the presence of biopolymers in the market is increasing every day, in the case of composite materials its use is still incipient [4].

Poly (lactic acid) (PLA) is a bio-based and biodegradable polymer with probably the most promising future among biopolymers [5]. The precursor of PLA, lactic acid, is obtained from the fermentation of carbohydrates and has different routes of polymerization [6]. The improvements in extraction and polymerization methodologies have produced a significant reduction in the cost of PLA and hence have increased its presence in the market. Moreover, PLA has higher mechanical properties than some commodity plastics leading to its use as a renewable replacement [4]. Nowadays, typical food packaging and containers are starting to be made of PLA [7]. Nonetheless, its mechanical properties need to be improved to replace engineering plastics. A possible solution is the formulation of composite materials. The addition of a phase with higher mechanical properties in the polymeric matrix led to stronger and stiffer materials [8].

In the case of oil-derived matrices, generally with lower mechanical properties than PLA, its reinforcement with inorganic fillers such as glass fibers (GF) has been a total success in industry and market. It is possible to obtain a huge variety of different applications and products nowadays. These composite materials represent around 98% of European composites [9]. Nonetheless, these composites show poor or negligible recyclability and the use of GF as reinforcement for biopolymer materials has limited interest [10]. To improve the sustainability of such materials, these inorganic fillers have been replaced by organic ones such as lignocellulosic fibers [11,12,13]. Although the use of these fibers reports some limitations, its use has a lot of benefits: low cost, renewable and recyclable resources, low densities, and the possibility of adding high reinforcement contents in the materials. Recently, because of the advances in bio-based and biodegradables polymer matrices, the objective of materials research has moved to the production of totally bio-based and biodegradable composites with tailored properties. In this sense, PLA is one of the most promising polymer matrices susceptible to be reinforced with natural fillers, due to its competitive mechanical properties. The high stability of PLA in normal conditions, regarding other bio-based and biodegradable polymer matrices, can enable its use in long-time applications. At the same time, its capacity to be degraded or composted avoids the problematic accumulation of composites material residues [7,14,15]. Moreover, it is interesting to remark on the environmental effect of these composites. A growing of environmental and life cycle analyses of bio-based matrices and blends and composites is observed in the past years. Generally, these studies showed a global potential warming of PLA similar to fossil matrices. This result can be reduced when the PLA plant and feedstock is well established until negative values [16]. Thus, a composite material using the same type of feedstock for its reinforcement only could enhance the sustainability of these materials.

Although the benefits of the use of a renewable and recyclable resource like lignocellulosic fibers as reinforcement, this kind of fibers show some limitations: difficulties in the dispersion of the reinforcement in the matrix due to the different polarities of the phases; an increment of the water uptake also related with the polarity of the fibers that can produce a reduction in the mechanical properties of the composite and limit its application; a limited range of process temperatures as the degradation of natural fibers starts around 200 °C [17,18]. However, PLA composites from cellulosic fibers have been produced previously showing interesting mechanical and thermal properties [19,20,21,22,23]. The PLA and bleached Kraft softwood pulp (BKSP) compounds have been studied from their behavior in terms of tensile strength, where the results revealed that lignin has a negative effect on the interface of PLA composite materials. As well as that PLA/BKSP composites do not have a perfect bonding system. However, BKSP showed good performance as a reinforcement for composite materials with a PLA matrix up to 30% reinforcement in the tensile and flexural strength [24]. The micromechanics of Young’s modulus was also evaluated, obtaining competitive values compared to those of polypropylene fiberglass composites [25]. The study of the flexural strength of fully biodegradable PLA and BKSP composites showed an increase in the flexural strength of materials with higher fiber content. The intrinsic flexural strength of the fibers was determined as well as its coupling factor showing values ranging from 0.18 to 0.16 when the fiber content was increased in the material. Coupling factors close to 0.2 are well-bonded systems. The results obtained for PLA composites reinforced the hypothesis of poor fiber-matrix interaction, but the theoretical approximations cannot be certain. The thermal properties of PLA and BKSP composites have also been studied, showing that BKSP diminished 40 °C the starting degradation temperature of the composites in comparison with PLA. Nonetheless, the glass transition temperature (*T*g) was slightly affected but the crystallization of the matrix was improved due to fibers’ presence. Moreover, the stiffness of the composites materials was superior to the matrix due to the fiber addition. A similar effect is observed in the thermal expansion coefficient where the composites showed lower values because of the restrictions of the fibers [23,26]. On the other hand, it has been found that other bleached fibers such as bleached kraft hardwood pulp fibers also exhibit similar behavior [20]. Nevertheless, the impact and water uptake behaviors of these composites are seldom evaluated. 

Impact resilience is a necessary property for many applications. Flexural and tensile properties evaluate the effect of a constant force that is slightly increased and represent only a part of the information needed to evaluate the use of materials under real conditions. Impact tests reproduce a real situation for many parts [27]. PLA has been usually modified in the literature to improve its impact resilience [17,28,29,30]. Nonetheless, in the case of composite materials, impact resilience has a high dependence on the phases and their interactions. Poor interactions between fibers and matrix are highly significant in impact properties. Moreover, that property is generally reduced due to the addition of fibers. The evaluation of that property can lead to a depth analysis of the interface and the stress transfer in the composite materials. Besides, the main objective is to obtain suitable and biodegradable composites materials thus inhibit the use of impact modifiers that not always are biodegradable or are highly expensive [30]. On the other hand, the hydrophilicity of cellulose fibers can have a big effect on the mechanical properties of composite materials. It could significantly reduce the lifespan of the composites materials in its applications [31,32]. L. Duigou et al. found a direct reduction of the mechanical properties regarding the water uptake [33].

The study of the water uptake and its kinetics is relevant for the use of these composite materials and its probable applications. Besides, the effect of the interface in water uptake is critical, as it could affect the kinetics and the permeation of the water in the composite material [34]. The literature agrees on the fact that the water uptake of natural fiber reinforced materials can be one of its drawbacks [35]. Natural fibers are hydrophilic and enable the absorption of water. The presence of water inside the composite has notable effects on the mechanical properties of the materials, usually downgrading such properties and decreasing the lifespan of the products [36]. Nonetheless, there are examples of the use of natural fibers reinforced polyolefin in automotive and product design. Thus, the industry can take advantage of these materials despite its water uptake behavior [37]. Then, if PLA-based materials show water uptake behaviors similar or better than polypropylene (PP)-based composites, its utility can be proved.

In this work, the impact resilience and the water uptake behavior were analyzed from composites of PLA and bleached Kraft softwood pulp. The composites were produced with the help of a dispersive agent for the fibers to avoid the difficulties produced by the different polarities of the phases [24].

## 2. Materials and Methods

### 2.1. Materials

Poly (lactic acid) (PLA Ingeos Biopolymer 3251D) supplied by Nature Works (Naarden, The Netherland) was used as a polymer matrix in this work. The cellulosic material used as reinforcement was bleached Kraft softwood pulp (BKSP) from pine and was provided by Torraspapel S.A. (Sarrià de Ter, Spain).

Diethylene glycol dimethyl ether (Diglyme) was used as a dispersant agent during the compounding process and was supplied by Clariant (Sant Andreu de la Barca, Barcelona, Spain). Dichloromethane was used as a solvent to dissolve the PLA matrix during the fiber recovery from the composite materials and was purchased from Scharlab (Sentmenat, Spain). Toluene, ethanol, *o*-toluidine blue, and sulfuric acid were also supplied by Scharlab (Sentmenat, Spain) and used for fiber characterization. Finally, methyl-glycol chitosan (MGCh) and potassium polyvinyl sulfate (PVSK) standard (N/400 concentration) (Wako Chemicals, GmbH, Neuss, Germany) were used for fiber’s polarity determination.

### 2.2. Methods

#### 2.2.1. Fiber Chemical Composition and Polarity

The chemical composition of reinforcing fiber was obtained following TAPPI standards: ashes (T211), extractives (T204), and soluble lignin (T222). Ashes were determined directly by burning fibers in the oven at 600 °C until the ashes were completely white. Extractives were extracted with a mixture of toluene: ethanol in a Soxhlet apparatus. The extractives can be obtained as the difference between the weights of the initial and the extracted samples. Extracted fibers were submitted to acid hydrolysis where lignin precipitates and can be filtered and recovered. Sugars are contained in the aqueous phase. The cellulose content was determined through anion exchange chromatography and considering the content of glucose as cellulose. Hemicelluloses were obtained from the difference between the other components. 

Fiber’s polarity was obtained by indirect titration using MGCh. A quantity of 4 mL of MGCh was directly added to a fiber water suspension of 1% w/w. The suspension was stirred 15 min and centrifuged at 3000 rpm for 15 min. A 10 mL aliquot of the supernatant was titrated with PVSK using the *o*-toluidine blue as an indicator [38].

#### 2.2.2. Composite Compounding and Samples Obtaining

Composite materials with reinforcement contents ranging from 10 to 35% of BKSP were produced in a Gelimat Kinetic mixer (Draiswerke, Mahwah, New Jersey, USA). The reinforcement and the polymer matrix were added in the mixing chamber at a low speed (300 rpm). Afterward, the speed was increased up to 2500 rpm. The blend was discharged when the temperature reached 195 °C. Finally, the blend was cooled down and pelletized in a knife grinder. 

**Figure 1:**
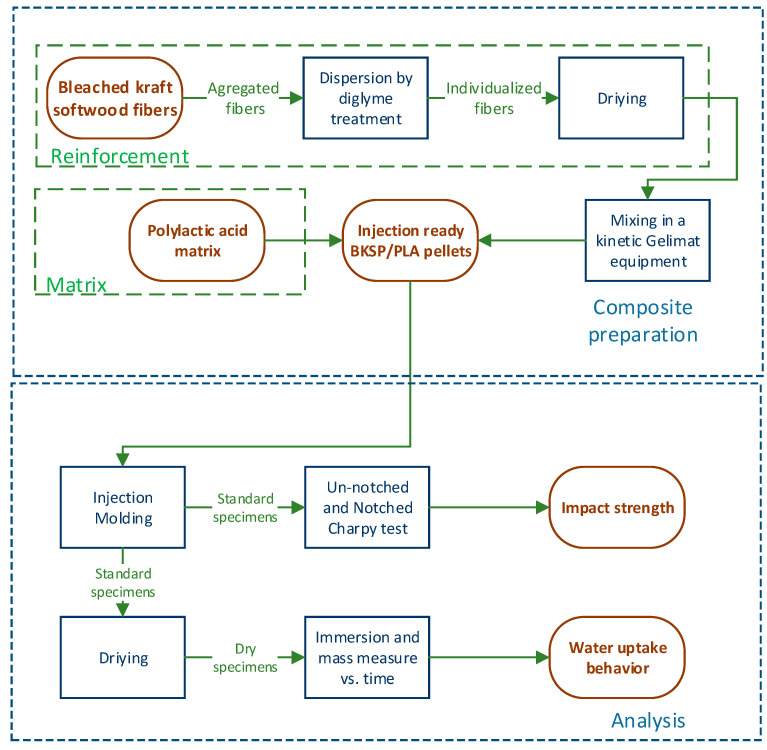
Schematic procedure of the proposed research.

The specimens for the impact test (Charpy, ISO 179) were obtained by injection molding using an Allrounder-220M injection molding equipment (Arburg, Eschweiler, Germany). The temperature profile was 180–190–200–210 °C and the pressure was increased with the fiber content with a maximum of 400 bars for the volumetric phase and 37 bars for the maintaining pressure phase. Figure 1 shows a schematic procedure of the materials and methods used during the research.

#### 2.2.3. Impact Test

The absorbed energy under impact was determined by Charpy impact test following the ISO 179 standard specifications. A Resil 5,5 hammer supplied by Ceast (Pianezza, Italy) was used for measuring the absorbed energy. At least five notched and unnotched specimens were prepared and tested. 

#### 2.2.4. Scanning Electron Microscopy (SEM) 

Micrographs of the fractured surface of the impact test samples were obtained by scanning electron microscopy (SEM). The equipment was a Zeiss DSM 960A (Carl Zeiss Iberia, Madrid, Spain). Samples were gold-coated before observation.

#### 2.2.5. Water Uptake Measurement 

The water uptake of composites was measured by an immersion test. Composite specimens were dried at 80 °C for 2 h to remove any residual moisture and then immersed in distilled water at 23 °C. Samples were kept under immersion until saturation. The water uptake was calculated by the difference by weight (*W*) from the initial weight (*W*_0_) of the samples (Equation (1)).

Water uptake (%) = (*W* − *W*_0_)/*W*_0_ × 100 (1)

#### 2.2.6. Preliminary Analysis of the Environmental Impact of the Composites

The analysis was based on the carbon footprint and the energy needed per kg of polymer and composite. The values were obtained from the environmental analysis database of SolidWorks^®^ and the literature [39,40,41,42].

## 3. Results and Discussion

### 3.1. Fiber’s Chemical Composition and Polarity Behavior

The chemical composition of BKSP is shown in Table 1. The chemical composition of mechanical pine pulp was used for comparison.

The extractives, ashes, and lignin contents of BKSP are low, all of them lower than 1%, because of the intensity of the chemical treatment (the Kraft process and the posterior bleaching). The removal of these components through the chemical treatments led to totally modified fibers with high cellulose contents. In comparison, the mechanical pine pulp used as a reference for unbleached softwood fibers shows a high content of extractives, ashes, and lignin. Moreover, these components are mainly situated on the surface of the fiber [43,44,45]. In the case of BKSF, the treatment removed these components from the fiber surface and increased cellulose availability. Besides, these components are generally undesired as their presence in the fiber surface inhibits the fiber–fiber and the fiber–matrix interactions [46]. Cellulose is a highly polar component with three primary hydroxyls per glucose unit. These hydroxyls can establish strong intermolecular H-bonds with other cellulose chains obtaining a strong intermolecular structure or network [47]. An example of the strength of these bonds is the case of brown and white paper, where white papers show higher resistances because of the higher capacity of the cellulose chains to establish H-bond interactions between bleached fibers due to the higher availability of cellulose in the surface and the lack of the other components. Nonetheless, while removing lignin, extractives, and ashes in the surface enhances the interaction with cellulose, in composite materials, it can be a disadvantage. Polymer matrices are usually apolar and its affinity with cellulose is poor. That poor affinity led to fiber aggregates and poor dispersion in the polymer matrices that reduce drastically the mechanical properties of the composite materials. It has been observed that a small quantity of lignin and extractives are adequate to improve the dispersion of the fibers in the matrix [48]. In this work, diglyme was used as a dispersing agent to improve the stress-transmission between fiber and matrix, obtain a correct dispersion of the fibers in the polymer matrix, and avoid fiber aggregates. Diglyme adheres to fiber’s surface during the fiber dispersion in water, blocking its polar groups. Once the fiber is drained and dried, the diglyme is evaporated and led to individualized fibers. The correct dosage of diglyme was previously studied in another work [24].

Although the dispersion negative effects of high content cellulose fibers such as BKSP can be solved with the use of a dispersing agent, the differences in polarity have a significant effect on the transmission of the stress. Components with high differences in polarity cannot establish strong interactions or enough of them to correctly transfer stress and can reduce the impact strength. The polarity of fiber and matrix is shown in Table 2.

The polarity values showed two close results indicating a similar interaction with the MGCh. The MGCh is a polar molecule, thus a higher interaction with the BKSP can be expected. Nonetheless, the polarity of MGCh was lower in comparison with cellulose fibers because of the presence of more apolar groups in the chain that can inhibit the interaction with highly polar groups. It is in concordance with the observed for mechanical softwood pulp treated by stone groundwood process (SGW) where fibers with high apolar groups in the surface (lignin and extractive) showed higher polarities measured with MGCh [49]. In that case, the polarity of BKSP is not appreciated, as the high density of hydroxyl groups on the surface of the fibers inhibit the interaction with the MGCh. Nonetheless, the result of PLA, although it is really low, indicates some kind of interaction with the polar components. These differences in polarity seem to indicate that probably an adequate interaction between fiber and matrix will not be obtained. The results are similar to those obtained with PP and bleached Kraft fibers composites [38,50]. In that case, and as it is generally known in the literature, a coupling agent is necessary to obtain some stable interaction between the matrix and the reinforcement. Moreover, this phenomenon is not only limited to cellulosic fibers, but it is also found when glass fibers are used as reinforcement. In GF-reinforced composites, the use of coupling agents or surface modifications is common [51,52].

### 3.2. Impact Strength

Composite specimens were submitted to notched and un-notched Charpy tests. Table 3 shows the obtained un-notched ($I_{U}^{C}$) and notched ($I_{N}^{C}$) impact strengths.

**Figure 2:**
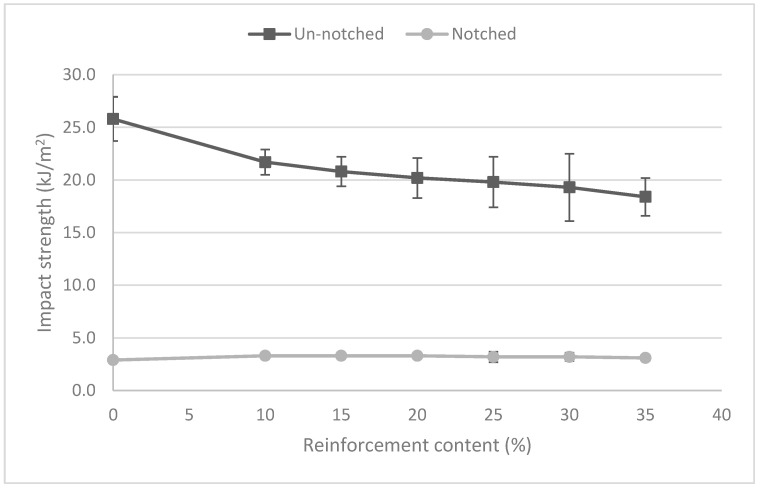
Evolution of the Charpy un-notched and notched impact strengths of the composites against BKSP content.

The results showed a clear effect of the reinforcement contents on the impact strength of un-notched specimens. These strengths decreased with increasing BKSP contents. This can be an indication of crack propagation through the interface due to poor matrix fiber adhesion [53]. This was expected regarding the results observed in polarity previously. On the other hand, the notched specimens returned almost the same value despite the reinforcement percentage. This result indicates a poor contribution of the reinforcements to the impact strength of the composites. In the case of weak interfaces, an increase of the notched impact strength is expected as fracture is already produced and the energy is devoted to fiber pull-out [53]. The decrease of the impact strength of un-notched composites can be related to the already observed increase of the fragility of the composites when submitted to the tensile and flexural tests, and a change from ductile to brittle fracture [17,21,23,54]. The strain at break, under tensile test conditions, of these composites decreased noticeably while the percentages of reinforcement were increased. This phenomenon had a clear effect on the toughness and resilience of the composites. The increasing fragility can be explained by the decreasing percentages of the PLA matrix, the continuous phase, and the increasing percentages of stronger but more fragile reinforcements. Other authors pointed out the increasing stress concentrations in the vicinity of the fiber [55] or the presence of too many fibers end within the body of the composites [56]. A gap between the impact strength of PLA and the composites can be observed in Table 3. The percentage decreases between the impact strength of the un-notched PLA specimen and the PLA/10%BKSP composite was 18.9%. The differences between such impact strengths of the materials with 10, 15, 20, 25, 30, and 35% wt BKSP contents were 4.3%, 2.9%, 2.0%, 2.6%, and 4.9%, respectively. The similarity between such percentage losses suggests a linear reduction of the un-notched impact strength of the composite materials (Figure 2).

Figure 2 shows the gap between the impact strength of the un-notched and notched samples of PLA and its composite materials. The figure also shows a linear evolution of the impact strength of the composite materials. A linear regression correlation between the un-notched impact strengths of the composites, against fiber volume fraction, returned the equation: $I_{U}^{C}$ = −13.107$V^{F}$ + 22.725, with a correlation factor R^2^ = 0.98. Similar behaviors have been reported for other natural fiber-reinforced composites [55,57,58]. This negative slope shows the decreasing contribution of the matrix to the impact strength of the composites. 

PLA/BKSP composites are presumably fully bio-based and biodegradable materials, as both phases are so. Commodity materials for the industry like GF-reinforced polypropylene (PP) composites are based on a polyolefin oil-based matrix and a mineral reinforcement that spends high amounts of energy to be produced. Such commodity materials have the trust of the automotive, aeronautic, and product design industries [59,60]. Thus, the properties of the PLA-based materials must be compared with commercial composites to assess their competitiveness in similar applications. The impact strength of un-notched PLA/BKSP composites is lower than PLA/GF composites, where a material with 30 wt % of glass fiber reaches 39.4 kJ/m^2^ [56]. Other authors have reported un-notched impact strengths of 72.24 kJ/m^2^ with a 30% Cordenka-reinforced PLA [27]. PP-based composites report better impact strengths, mainly because of the higher toughness of PP in front of PLA [56,61]. The higher toughness is highly related to the differences in polymer chemistry. PLA has a rigid chemical structure while PP carbons have a high free rotation. In PLA, the ester functional group is based on atoms bonded with each other forming a plane, limiting the rotation of the carbon in the aliphatic chain, leading to a rigid structure. This rigid structure harms the dissipation of the energy absorbed in the elastic impact and limits the wettability of the fiber by the polymer matrix. The maximum fiber content included in PLA composites is around 35–40% while other polymeric matrices can incorporate fiber contents up to 50%. Another property affected by the polymer chemistry is the *T*g, around −10 °C for PP, and around 60 °C for PLA. These differences play a major role in the impact strength results as it is the temperature where the amorphous phase of polymers starts to move. In the case of PP, the amorphous fraction of the polymer has high mobility during the test, which is performed at 23 °C. In the case of PLA, the testing temperature is under its *T*g, and it is one of the reasons for the poor impact resistance of PLA. Anyhow, the literature places at 30 kJ/m^2^ the impact strength of materials used in the automotive industry [27]. Although the literature shows a big amount of data on the impact properties of composite materials, the authors preferred to prepare GF-reinforced PP composites to reproduce the same testing procedures. It is known that to increase the mechanical properties of PP/GF materials, adding a coupling agent like polypropylene functionalized with maleic anhydride (MAPP) has a noticeable impact, and is common in the formulation of the commercial materials. Based on previous works, uncoupled and coupled material batches were prepared [20,21,25]. The coupled composites added 6% of MAPP against reinforcement content. Table 4 shows the un-notched impact strength for the uncoupled or sized GF ($I_{\mathrm{sized}}^{C}$) and coupled (ICcoupled) PP/GF composites.

**Figure 3:**
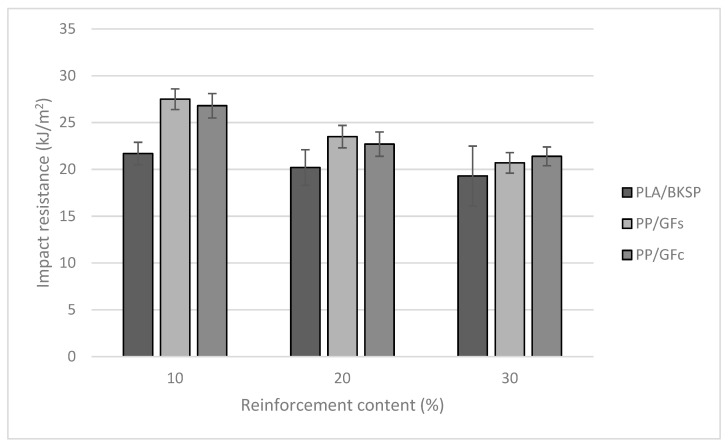
Evolution of the Charpy un-notched impact strength of PLA/BSKP and uncoupled and coupled PP/GF composites against reinforcement weight.

PP/GF composites decreased its impact strength with the increasing amounts of reinforcement. Unlike PLA, PP did not break under the test conditions as the used Charpy pendulum was not able to produce enough energy to break the specimen and just could deform the specimen. This shows the initial advantage of PP-based composites when tested under impact conditions, as PLA is more brittle than PP. The impact strength of the coupled composites is higher than the uncoupled, showing the effect of strong interfaces on such property. Nonetheless, as the percentage of reinforcement increased, PLA/BKSP materials tend to reach similar or superior impact strengths. Figure 3 compares the un-notched impact strengths of PLA/BKSP and, uncoupled and coupled PP/GF composites with fiber reinforcement contents ranging from 10 to 30%.

**Figure 4:**
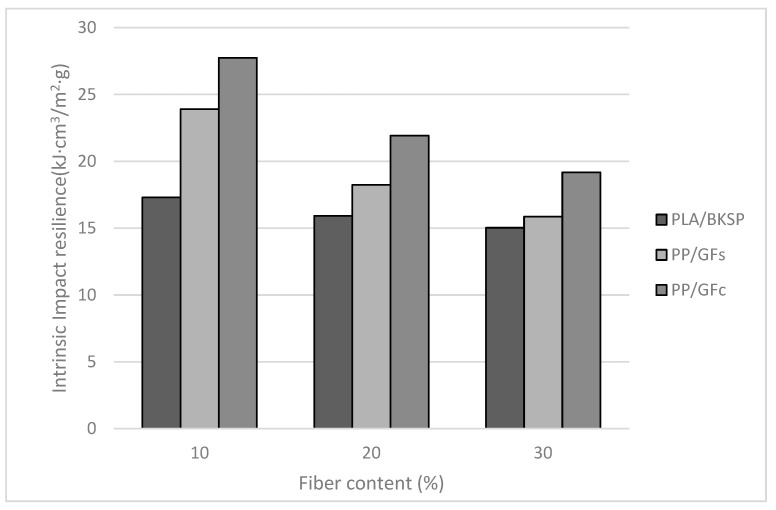
Evolution of the Charpy un-notched specific impact strength of PLA/BSKP and uncoupled and coupled PP/GF composites, against reinforcement weight contents.

Figure 3 shows how in high contents of BKSP, PLA/BKSP composites showed similar or better impact strengths than PP/GF composites. These results can be related to the highest brittleness of GF regarding BKSP [62,63,64]. Although the slope of the PP/GF composites was higher than PLA/BKSP, coupled PP/GF composites always showed higher impact strengths than PLA/BKSP. On the other hand, a composite with a 20% wt BKSP content shows a higher impact strength than any PP/GF sized composite with sized GF contents superior to 20% wt. Thus, it is possible to prepare composite materials that add higher amounts of BKSP, a cheaper phase than PLA, and more sustainable than GF, and compete with commercial PP/GF materials. Comparing the impact strengths of the composites against its reinforcement volume fractions it can be seen how for volume fractions around 0.1, the PLA-based composites showed similar impact strengths than the PP-based ones. This is due to the comparatively higher density of GF in front of BKSP. Nonetheless, PP-based composites continue showing lower densities (Table 1 and Table 2). To subtract the different densities of the composites, Figure 4 compares the specific impact strengths of the materials against reinforcement content.

The figure clarifies that only the PLA/BKSP composites with 30% wt reinforcement contents or higher can compete with uncoupled PP/GF composites in terms of net contribution of the reinforcements to the impact strength of the materials. Despite the natural fibers being lighter than GF, the density of the PLA in front of PP affects noticeably the specific property of its composites. Nonetheless, the high tensile and flexural properties of PLA/BKSP composites can equalize these disadvantages [20,21,25]. Every case must be studied in detail and the advantages of fully biodegradable and bio-based materials in front of non-biodegradable materials based on perishable resources must be part of the decision.

**Figure 5:**
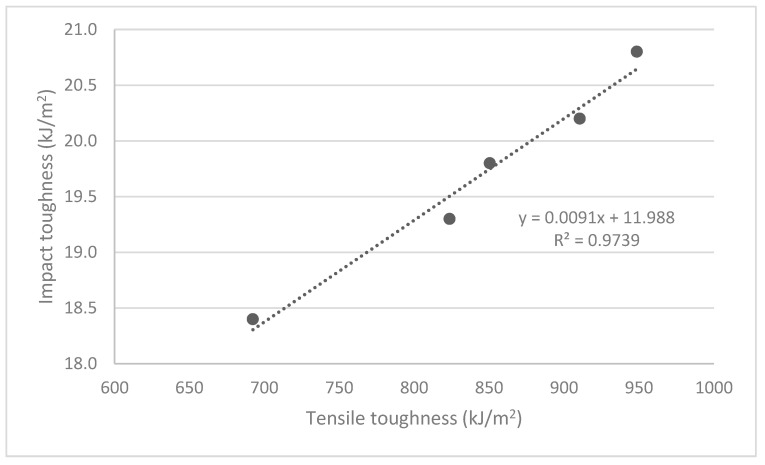
Correlation between the toughness of the composites obtained from tensile and impact tests.

In previous research, part of the authors computed the toughness of the composites as the area below the stress-strain diagram [24]. Figure 5 explores the correlation between the data obtained from the impact and tensile tests.

Figure 5 shows the high correlation between both values (R^2^ = 0.97). Although, the toughness measured as the area below the stress–strain curve is more of a qualitative nature than impact toughness, a certain correlation was expected.

Notched specimens showed different behavior. There was a 12% increase of the notched Charpy impact from the PLA matrix to the composite with a 15% wt BKSP content, but then this impact strength remained almost unchanged for the rest of the composite materials. In the case of weak interfaces, notched impact strength increases with the reinforcement content as the area of the fracture increases because of the energy absorbed by fiber pullout [55]. Strong interfaces promote a brittle fracture with crack propagation in all the phases. In these cases, the notched impact strength tends to decrease with the amount of reinforcement [53]. The obtained values are similar to PLA/Abaca composites, with 3.7 kJ/m^2^, but inferior to cellulose and glass fiber 8.2 and 10.1 kJ/m^2^, respectively. The main reason can be found on the regular surface and aspect ratio of glass fiber that increases the energy needed to pull out the fibers from the matrix [65].

During the Charpy test, there are two main phenomena related to the impact strength. On the one hand, there is the creation of the fracture, with the corresponding energy, and on the other hand, the energy needed to propagate such fracture. Thus, the energy necessary to break a sample can be deduced from the following equation:

w = w_i_ + w_f_ + w_m_ + ∑w_fm_ (2)

where w is the amount of energy needed to break the sample, w_i_ the energy necessary to start a critical fracture, w_f_, and w_m_ the energies to break the fiber and the matrix, respectively. Finally, w_fm_ is related to fiber–matrix interface related phenomena, like fiber sliding or pullout [55,58,66]. In the case of the notched samples, w_i_ disappears, as the notch provides a critical fracture and the impact adds only the energy needed to propagate such fracture. Thus, the differences between Charpy un-notched and notched impact strength amount for w_i_. Table 3 shows such values. It was found that the energy needed to create the fracture decreased with the amount of reinforcement. This decrease was noticeable between the PLA and the composites, but its value only decreased slightly with the increasing amounts of reinforcement.

When a fracture is initiated, such crack tends to expand among the most brittle phase, either the matrix, the fiber, or the interface. Usually, the interface is the weakest phase on a natural fiber-reinforced composite [55]. As commented above, the energy needed to propagate the fracture tends to increase with the amount of reinforcement. This is related to the fact that the increasing presence of fibers in the fracture surface increases the length of the total fracture, as this fracture has to surround such fibers or increases the energy needed to pull out the fibers from the matrix [67]. Nonetheless, the PLA/BKSP composites showed almost the same energy to propagate the fracture, with increasing amounts of reinforcement. The initial increase can be related to the increased length of the fracture, but its decrease with the percentage of fibers can only be due to a weak interface.

**Figure 6:**
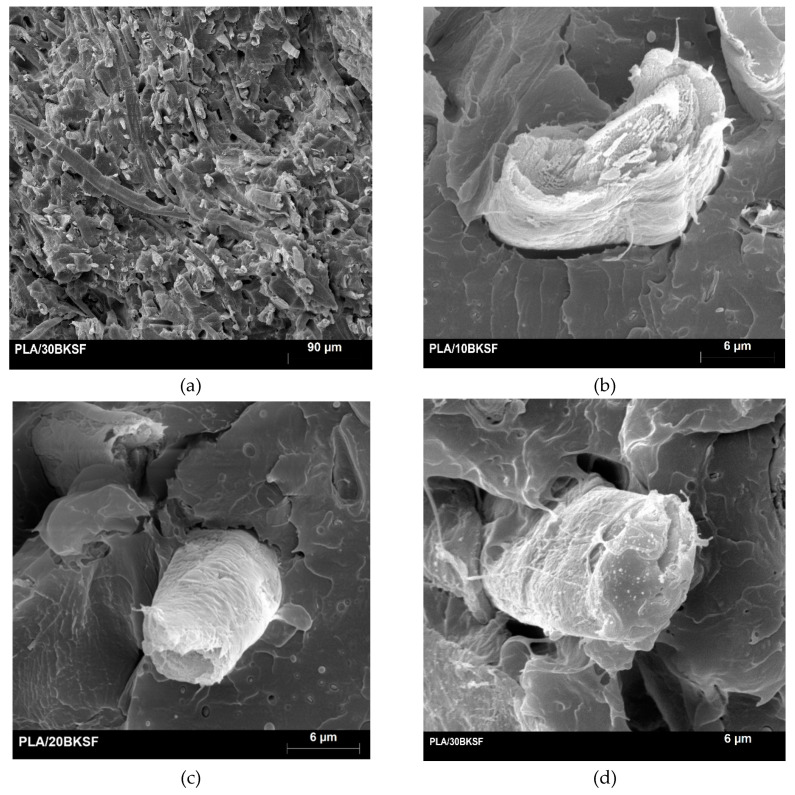
SEM micrographs of the fractured sections; (**a**,**d**) from a composite with a 30 wt % of BKSP; (**b**) from a composite with a 10 wt % of BKSP; (**c**) from a composite with a 20 wt wt % of BKSP.

Figure 6 shows a micrograph of the fractured surface.

Figure 6a shows a general view of the section. There it can be seen a good dispersion of the fibers, without visible fiber bundles. The figure shows also some pull-outs and voids. Some fibers show almost horizontal orientation, agreeing with the semi-aligned nature of the composites. Figure 6b–d show details of a single fiber inside composites at BKSP contents ranging from 10 to 30 wt %, respectively. There are no noticeable differences between the cases. The figures show pulled out fibers, and Figure 6c the end on another fiber. The hypothesis for the fracture propagation indicates weak interfaces, based mainly on mechanical anchorages of the fibers to the matrix. Moreover, the surface of the fibers has low roughness due to the chemical treatment and bleaching, hindering the mechanical anchoring and no matrix remains can be observed, a signal of few chemical bonding between such phases. Thus, a pullout of the fiber can be assumed. The other fiber is partially wrapped by the matrix but the poor adhesion to the matrix is also clear. A possible granular fracture of such fiber is possible, but this fiber shows a low section. The crack propagates all around the interface and from one fiber to the other as can be observed in the figure. The fibers stop the propagation of the fracture creating irregular ruptured surfaces of the PLA near the reinforcements. In this point, a secondary fracture propagation due to fiber pullout takes place [68]. The strength of the interface hinders a possible propagation of the fracture through the fiber. The decrease of the $I_{N}^{C}$ when the percentage of reinforcement increases can be due to the decrease of matrix content and the increase of fiber easily pulled out from the matrix.

To increase the impact strength of PLA/BKSP composites, the aspect ratio of the fibers must be improved, to increase the pullout length of the fibers, and the strength of the interface must be also tuned, to maximize the energy expended during fiber pullout.

### 3.3. Water Uptake Behavior

Water uptake of polymers and composite materials has a significant effect on its mechanical performance. Water is a small molecule that can easily diffuse in solids materials. Nonetheless, in the case of polymeric materials, the penetration of water inside the polymer phase produces a disruption in the spatial disposition of the chains, inhibiting the interaction between them, and displacing the chains [69]. It causes a swelling of the material and a loss of mechanical performance. Moreover, some polymers, like in the case of PLA, can be fully degraded by water under certain conditions, which led to another disadvantage of the use of these polymers in certain applications [70]. Regarding natural fiber-reinforced composite materials, the effect of water is enhanced as the hydrophilic behavior of natural fibers favors the mobility of water inside the material while the fibers became swelled. The swelling of the fibers can also produce the crack of the material [18,71].

**Figure 7:**
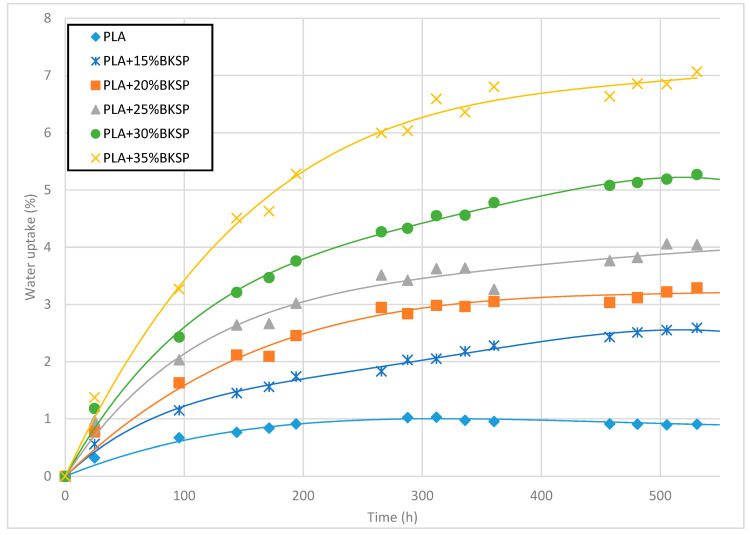
Water uptake behavior of PLA and PLA-BKSP composites.

The water uptake test of PLA composites was performed in an accelerated form by immersing it in fluid at room temperature (23 °C). The temperature was controlled by a climatic chamber to prevent any change in temperatures. Water uptake kinetics and degradation are sensible to this parameter. Figure 7 shows the behavior obtained for pure PLA and its composites.

The results showed a significant increment of the water weight in the materials with the time. The water uptake at the saturation point (M_∞_) of the virgin PLA was 0.94% and the sample was almost saturated in the first week of immersion. The results are in concordance with the observed in the literature for neat PLA [22,70,72]. The M_∞_ is related to the interactions between the polymer matrix and the water while the saturation time is related to the kinetics and thickness, or in other words to the ability of water to penetrate the material. The M_∞_ is higher than the observed for polyolefin like PP or PE [19,55,73], where a small water uptake is observed because of the high apolarity of the aliphatic polymer chains. PLA is not only composed of carbon–carbon and carbon–hydrogen bonds, as its main functional group is the ester group (–COOR). The presence of these groups produces a slight enhancement of the polarity of the matrix because of the oxygens in the chain. It is also observed in the water contact angle of PLA which is almost 25° lower than that observed for PP [74,75]. Nonetheless, the water uptake of PLA is significantly lower than the observed for polar polymer matrices like the case of polyamides, where water has strong chemical interactions, like H-bonds, with the polymer [76,77].

**Figure 8:**
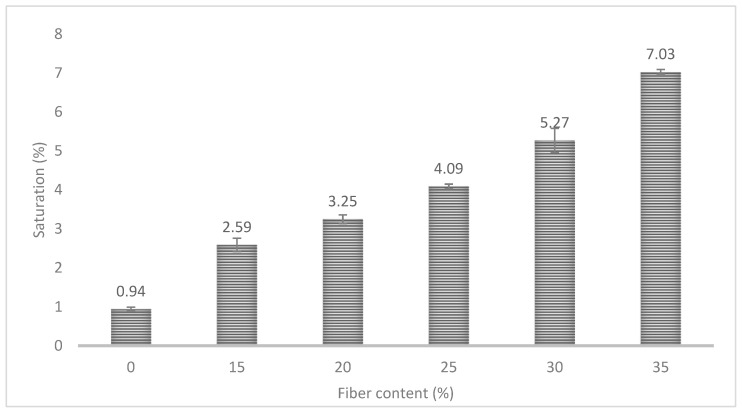
Water uptake of PLA and PLA-BKSP composite materials in the saturation stage.

However, the increment of the hydrophilic phase, BKSP, in the material represented a significant increment of the water uptake of the samples (Figure 8). The M_∞_ increased up to 7% when 35% of fiber was included as reinforcement. Moreover, the saturation stages were delayed up to 20 days of test for some samples. The increment of the water uptake was expected because of the introduction of a highly hydrophilic phase in the composite material but when the results are compared with cellulose-reinforced PP composites, the values were similar to those obtained for uncoupled formulations [19,78]. This agrees with that observed previously in the impact resistance, as a poor fiber–matrix interaction can enhance the penetration of the water through the material [78].

Kinetics of the water uptake in polymeric materials can be modeled using the linearized equation of the Fick law (Equation (3)):

(3) $$log\left(M_{t}/M_{\infty }\right)=n\;log\left(t\right)+logK$$

where *M*_t_ is the saturation value at a certain time (*t*). The parameter *n* and *K* are the Fick’s constants directly obtained and referred to the type of water diffusion case described in the Fickian model [11]. The *n* parameter is a parameter that indicates the mobility of the water in comparison with the polymer chains while the *K* constant is related to environmental conditions. Table 5 shows the results obtained for PLA and its composite materials.

The *n* parameter of all the formulation is near 0.5 indicating a Fick’s diffusion case or also known as case diffusion I. A Fickian case is usually obtained in semi-crystalline polymers in which no strong interaction between the polymer and the water is observed. In this case, the equilibrium inside the polymer is easily and fast reached as observed in the PLA. Composite materials showed a higher saturation point due to the capacity of fibers to absorb water. It can be the reason why the *n* parameter is slightly decreased for almost all the samples, as also the fiber absorption is taken into account in the kinetics and the polymer chain mobility is most restricted to fiber presence than in neat PLA. Nonetheless, the values are around 0.5 and similar results have also been observed in PP-cellulose composites [55,73]. The *K* value is related to the environment of the samples. Almost all the formulations gave rise to a similar value, as it was expected, because of the same environmental conditions, except for the sample reinforced with 35% of BKSP. However, the *n* parameter of the 35% fiber-reinforced composite was higher than the expected. The results could indicate a non-totally well-injected sample with some porosity that could affect the test resulting in a slightly different tendency. A 35% fiber is a high fiber content and it could be difficult to obtain correct injected samples although visually defects were not observed.

When a Fickian case has been observed in the material, the diffusion coefficient (*D*) can be obtained from the Equation (4) for relations of *M*_*t*_/ *M*_∞_ lower than 0.5 and considering the width of the sample (*L*).

(4) $$\frac{M_{t}}{M_{\infty }}=\frac{4}{L}\cdot {\left(D/\pi \right)}^{1/2}\cdot t^{1/2}$$

**Figure 9:**
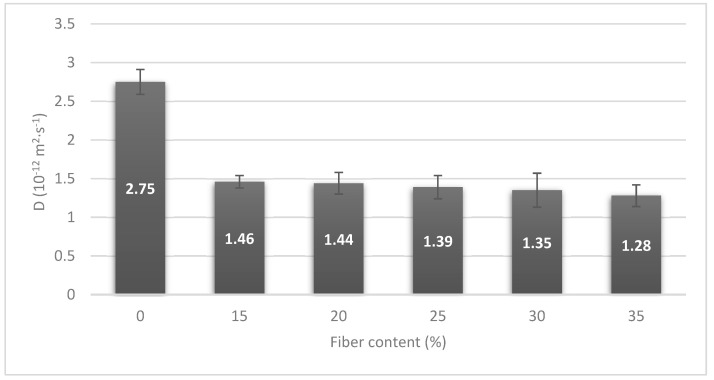
*D* parameter calculated in composite materials regarding fiber content.

*D* coefficients of PLA and its composite materials are represented in Figure 9. The diffusion coefficient indicates the facility of water to penetrate the material. Higher capacity is observed in neat PLA than in the case of composite materials. The presence of fiber reduced the *D* coefficient to the half for composite materials indicating a higher difficulty of water to penetrate these materials. These results can be related to the stiffness of the fibers which makes the polymer chain mobility difficult and increases the saturation time in samples with a similar width. Nonetheless, the increment of the fiber content has a small effect on the *D* coefficient, as samples showed similar values in all the composite materials with a maximum difference of 0.18 between the 15 and 35% of fiber content. The difference value is similar to the error obtained in the test, so it is not a representative difference. It seemed that the increment of the fiber content had a lower effect than the presence of fibers.

As above mentioned, PLA can be degraded in water in certain conditions. Generally, it has been found that PLA is degraded by water when the temperature is above its *T*g temperature [79]. The *T*g of PLA is around 60 °C, as previously mentioned, and the presence of the fibers did not induce to have a significant effect on this temperature transition [26]. Thus, at the testing temperature, any degradation was expected. A parallel test to the water uptake was carried on to determine the influence of fibers on the degradation of the composite at room temperature. Specimens were immersed in the same conditions of water uptake and at a certain time the samples were removed and dried to evaluate the difference in weight. Neat PLA samples did not show any weight loss as it was expected. In the case of composite materials, a small reduction in weight was observed. However, losses ranged between 0.2 and 0.9%, obtaining a higher loss when the fiber content is increased in the material. It seemed as fiber can have some effect on the hydrolytic degradation of PLA chains. Gil-Castell et al. obtained lower molecular weights of PLA raising the fiber content in the PLA composites [72]. Nevertheless, the temperature of the test was over the *T*g. The small weight loss could be also related to a loss due to the swelling of the fiber which can produce cracks in the matrix and producing a non-appreciable loss. More research is required to define the effect of the fiber in the degradation at low temperatures.

### 3.4. Preliminary Analysis of the Environmental Impact of PLA-Based Composites in Front of GF Reinforced PP Materials

Previous researches proved that natural fiber-reinforced PLA composites can reach similar properties as GF-reinforced PP composites. The tensile and flexural strengths strength of such materials were comparable [21,25]. Moreover, the stiffness of PLA and PP-based composites could be also equivalent [25]. It is important to cite that competitive tensile and flexural properties were reached without adding any coupling agent while PP-GF composites are required to achieve such values. Thus, PLA-based composites in addition to being based on renewable resources agree with the principles of green chemistry [21,23,24,80]. 

The resilience of PLA has been a concern for researchers, and as a proof, there are a large number of papers devoted to increasing such property [81,82,83]. These papers have proved different strategies to upgrade the resilience of PLA. Nonetheless, the authors show how BKSP-reinforced PLA composite can show impact strengths in line with GF-reinforced PP composites. Besides, the water uptake behavior of PLA composites is in line with other natural fiber-reinforced PP composites [55,76,78], reinforcing the suitability of such materials to be a greener alternative. Some of these composites are actually in use in the automotive and industrial design industries [84,85,86].

**Figure 10:**
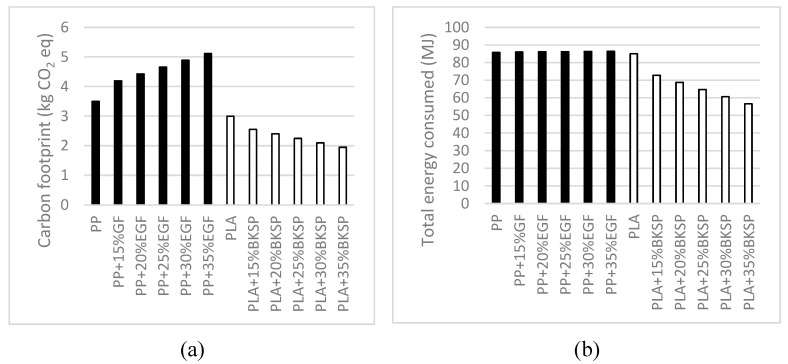
Environmental impact of GF-reinforced PP and SGW-reinforced PLA composites per kg of material production; (**a**): carbon footprint, (**b**): total energy consumed.

The industry has a lot of inertia and it is difficult to change its confidence in commodity materials like GF-reinforced PP composites. Nonetheless, some countries are evolving their laws in an intent to diminish the environmental impact of its industry. Thus, some industries must search for replacements of oil-based materials. Such materials must be based on renewable sources and show environmental impacts lower than oil-based materials. Figure 10 shows some of the more common environmental impact measurements for GF-reinforced PP and BKSP-reinforced PLA composites.

In a first view of the results, it is clear that PLA-based composites show lower impact values than PP-based materials. It can be seen that the carbon footprint needed to produce PLA are inferior to PP, and the energy consumed to produce both polymers are similar [39,40]. The carbon footprint reported values are in the range from 0.8 to 3.5 and 0.1 to 3.0 kg CO_2_ eq/kg polymer for the PP and the PLA, respectively [40]. The authors have adopted the maximum values to perform the analysis. On the other hand, the energies needed to produce a kg of PP or a kg of PLA are very similar. The literature shows values in the range from 41 to 85 and 25 to 85 MJ/kg polymer, for the PP and PLA, respectively [39,40]. Some authors’ values are as low as 0, but are controversial [39,40]. The highest deviations are observed when the reinforcements are added to the composites. On the one hand, the carbon footprint of GF is higher than PP and increases noticeably the carbon footprint of the composites [39]. On the other hand, the carbon footprint of BKSP is lower than PLA, and thus, the carbon footprint of the composites decreases with the percentage of reinforcement [41]. 

As commented, the energies needed to produce PP and PLA are similar. Nonetheless, the energy needed to produce GF is similar to PP, but the energy needed to produce BKSP is much lower than PLA [39,40,41]. Thus, PLA-based composites need less energy than PP-based materials for their production. In terms of sustainability, PLA-BKSP composites clearly showed an improvement in terms of carbon footprint and energy consumption.

## 4. Conclusions

The chemical composition, the polarity, the impact resilience, and the water uptake of BKSP-reinforced composites were analyzed in this work. The chemical composition of the fibers showed a fiber with high cellulose content that can make the interaction with the matrix and its dispersion in the matrix difficult. A dispersion agent was required to assure an adequate dispersion. Nonetheless, the polarity results were low for both compounds, fiber and matrix, which could indicate a similar behavior with adequate interaction. However, it is possible that only in the case of the matrix, the low result is directly related to a low polarity. In the case of the fiber, the result is covered up because the higher the polarity of the cellulose, probably the difficult the surface adhesion of the MGCh. The impact resilience of un-notched samples decreased linearly with the addition of fiber in the composite materials. These results were expected because of the reduction of the though phase by increasing the fiber contents and the presence of fiber ends. Nonetheless, the results were competitive enough in comparison with PP-GF composite materials. Moreover, PLA-BKSP composites showed a lower reduction of impact resilience than PP-GF composites. On the other hand, notched samples of composite materials showed an enhancement of resilience regarding pure PLA. The results were expected as normally the impact strength of notched samples increases because of the higher energy devoted to fiber pullout. The energy necessary to initiate the fracture can be estimated from the differences between the impact strengths of notched and un-notched samples. This energy is reduced when the fiber content in the composite material is increased and the results are related to a weak interface which is in concordance with the observed in the chemical characterization.

Water uptake of PLA showed a value of 0.94% which was increased up to a maximum of 7.03% for the composite material with 35% of fiber content. The increment in the saturation stage value of composite materials was expected because of the presence of BKSP that are highly hydrophilic components. Nonetheless, the saturation point was shifted to longer times in composite materials. Kinetics of the water uptake process was evaluated by Fick law. All the materials showed a Fickian case, with an n value close to 0.5. In the case of the *K* constant, the value was almost the same for all the cases except for PLA/35%BKSP, but the differences can be related to a not correctly compounded material. *D* coefficient was also calculated from water uptake curves. A significant reduction of the *D* coefficient was observed by the addition of fiber in the polymer matrix although the fiber content seemed to have a low impact on it. Finally, a degradation lower than 1% was found in PLA composites that were not observed for pure PLA.

A preliminary environmental analysis of the materials shows a clear advantage of the bio-based composites in front of commodities like GF-reinforced PP. Given the possible regulations that prevent or limit the use of oil-based material, PLA/BKSF composites are a robust alternative.

## Figures and Tables

**Table 1:** Chemical composition of bleached Kraft softwood pulp (BKSP).

	Ashes (%)	Extractives (%)	Lignin (%)	Hemicellulose (%)	Cellulose (%)
Bleached Kraft Softwood Pulp	0.34 ± 0.09	0.11 ± 0.05	0.87 ± 0.14	14.56	84.12 ± 1.79
Mechanical Softwood Pulp	1.64 ± 0.12	2.04 ± 0.07	28.96 ± 0.27	12.30	55.06 ± 1.84

**Table 2:** Polarity values of BKSP and PLA.

	Polarity (µeq MGCh/g Sample)
Bleached Kraft Softwood Pulp	8.5 ± 0.2
Stone Ground Wood	31.4 ± 0.1
Poly (lactic acid)	2.9 ± 0.2

**Table 3:** Impact resilience of Charpy un-notched and notched poly (lactic acid) (PLA)/BKSP composites against reinforcement content.

Sample	$V^{F}$	$\rho^{C}$ (g/cm^3^)	$I_{U}^{C}$ (KJ/m^2^)	$I_{N}^{C}$ (KJ/m^2^)	$I_{U}^{C}-I_{N}^{C}$ (KJ/m^2^)
PLA	0	1.240 ± 0.016	25.8 ± 2.1	2.9 ± 0.2	22.9
PLA/10%BKSP	0.090	1.254 ± 0.021	21.7 ± 1.2	3.3 ± 0.1	18.4
PLA/15%BKSP	0.135	1.261 ± 0.011	20.8 ± 1.4	3.3 ± 0.1	17.5
PLA/20%BKSP	0.181	1.269 ± 0.006	20.2 ± 1.9	3.3 ± 0.2	16.9
PLA/25%BKSP	0.228	1.276 ± 0.009	19.8 ± 2.4	3.2 ± 0.5	16.6
PLA/30%BKSP	0.275	1.284 ± 0.013	19.3 ± 3.2	3.2 ± 0.4	16.1
PLA/35%BKSP	0.323	1.292 ± 0.017	18.4 ± 1.8	3.1 ± 0.3	15.3

**Table 4:** Specific impact resilience of Charpy un-notched PLA/BKSP composites and uncoupled and coupled polypropylene (PP)/glass fibers (GF) composites against reinforcement content.

Sample	$V^{F}$	$\rho^{C}$ (g/cm^3^)	$I_{sized}^{C}$ (KJ/m^2^)	$I_{Coupled}^{C}$ (KJ/m^2^)
PP	0	0.905 ± 0.022	-	-
PP/10%GF	0.039	0.966 ± 0.031	23.1 ± 2.8	26.8 ± 3.1
PP/20%GF	0.084	1.036 ± 0.016	18.9 ± 1.3	22.7 ± 2.3
PP/30%GF	0.136	1.116 ± 0.043	17.7 ± 1.5	21.4 ± 2.0

**Table 5:** Fick’s constants calculated from the experimental results in PLA and its composite materials.

Fiber Content (%)	$M_{\infty }$ (%)	*n*	*K*
0	0.94 ± 0.05	0.51 ± 0.06	0.068 ± 0.013
15	2.59 ± 0.17	0.43 ± 0.09	0.067 ± 0.019
20	3.25 ± 0.11	0.47 ± 0.01	0.058 ± 0.002
25	4.09 ± 0.06	0.44 ± 0.02	0.063 ± 0.005
30	5.27 ± 0.31	0.45 ± 0.02	0.064 ± 0.008
35	7.03 ± 0.06	0.54 ± 0.01	0.040 ± 0.002
